# Intraoperative positioning related injury of superficial radial nerve after shoulder arthroscopy – a rare iatrogenic injury: a case report

**DOI:** 10.1186/1757-1626-1-47

**Published:** 2008-07-18

**Authors:** Vinay K Singh, Pankaj K Singh, Amir Azam

**Affiliations:** 1 Department of Trauma & Orthopaedics Surgery, Luton and Dunstable Hospitals NHS Foundation Trust, Luton, LU4 0DH, UK; 2 Department of Neurosurgery, Royal Hallamshire Hospital, Sheffield, S10 2JF, UK; 3 Department of Trauma & Orthopaedics Surgery, Manchester Royal Infirmary, Manchester, M13 9WL, UK

## Abstract

Arthroscopy of the shoulder is a well-established and routine procedure. The role is expected to increase further with an ever-increasing list of indications reflecting the gratifying results. Intraoperative injuries of nerves related to positioning are known but, fortunately, rare with shoulder arthroscopy. Appropriate assessment of patient and careful positioning is paramount in prevention of these injuries. Despite robust preventive measures, these injuries continue to occur from time to time. Although there are few reports of position-related intraoperative nerve injuries associated with shoulder arthroscopy, the involvement of superficial radial nerve (SRN) has never been described before. We report a rare case of positioning related injury of SRN in a 35-year-old female after arthroscopic rotator cuff repair and discuss the preventive and legal aspects. To authors' knowledge this is the first reported case of intraoperative positioning related injury of SRN.

## Introduction

Iatrogenic injury to a nerve during perioperative period is a well-known complication [1,2]. Any surgical procedure requires well-orchestrated handling of the patient by the team in order to avoid these injuries. Adequate patient assessment, preoperative planning and necessary training of staff are imperative [1]. Preexisting medical condition such as diabetes, peripheral vascular disease and malnutrition predispose to the increase risk of injury [1]. Most of these injuries are fortunately reversible and recover completely if diagnosed and managed appropriately [1]. Although mostly reversible, they still cause unnecessary disability and anxiety to the patients and may result in long-term morbidity, as illustrated by this case.

## Case presentation

**Figure 1:**
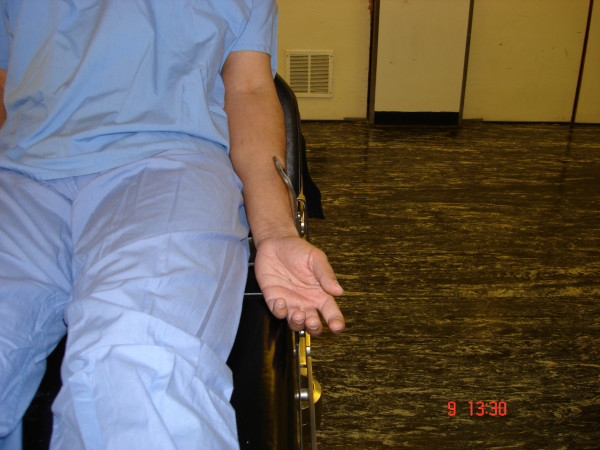
Simulated position of arm during arthroscopy in beech-chair position.

**Figure 2:**
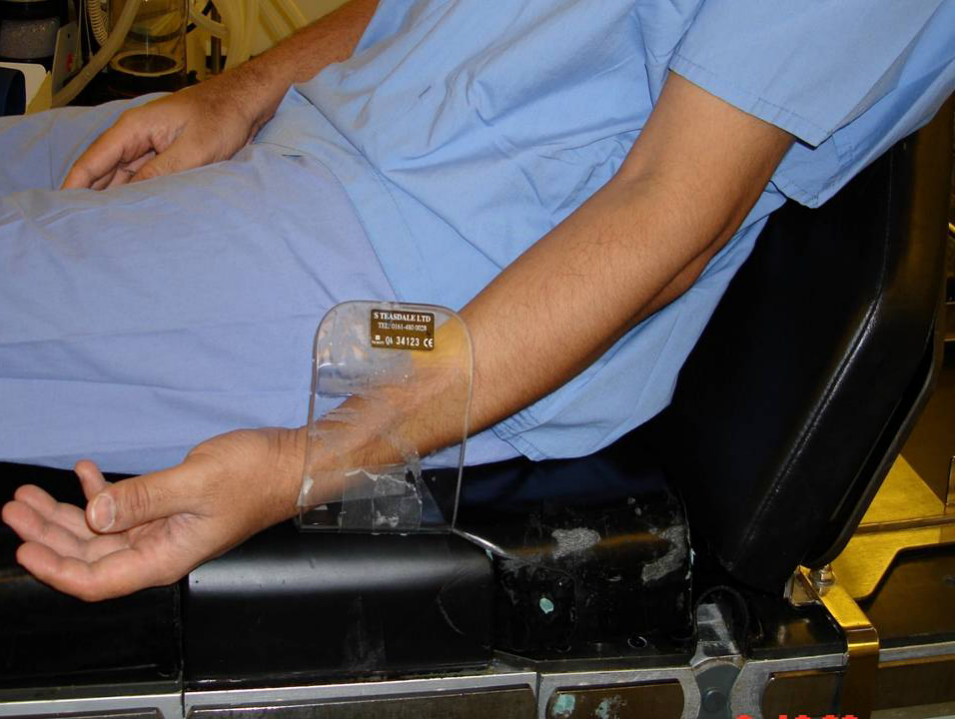
Simulated position of arm during arthroscopy in beech-chair position.

A 42-year-old female was admitted as a day case for an arthroscopic rotator cuff repair of right shoulder. Her main complaints were pain and stiffness in right shoulder for past two years. She had surgery in beech-chair position with operated side supported by arm support (Figure 1 &2). Surgery was performed under interscalene block and operative time was around 2 hours. She was an otherwise fit and healthy female with no active medical problems. Postoperatively, she complained of pins and needles with paresthesia on outer aspect of lower forearm and dorsum of hand in anatomical snuffbox on operated side. She had isolated sensory deficit corresponding to SRN distribution with no motor deficit. Rest of the neurological examination and cervical spine was unremarkable. Her symptoms were thought to be originating from the nerve block and she was reassured and discharged. In her two weeks follow up she still complained of persistent symptoms. There were no changes in her examination findings. She was reassured that her symptoms should improve with time. In three-month follow-up her shoulder was better but still had paresthesia in SRN distribution. The anesthetic team in-charge was contacted for advice, and they felt that her symptoms could not be attributed to as a complication of interscalene block. Nerve conduction studies were requested to identify the lesion. Even after six months her symptoms did not improve. Nerve conduction studies confirmed isolated lesion of SRN 18 cm proximal the radial styloid with positive Tinel's sign at the same level. After excluding all the possible causes and retrospective analysis, a diagnosis of positioning induced pressure injury of SRN was made. The patient was managed conservatively as per advice of the neurologist. In two-year follow-up the pins and needles have settled but she is left with residual decreased sensation on dorsum of hand in SRN distribution.

## Discussion

Positioning related nerve injury is well known complication of surgery but fortunately it is rare [1]. It is commonly seen to involve brachial plexus, sciatic, tibial, common peroneal, ulnar, radial, median and axillary nerve [1]. It may also involve musculocutaneous, long thoracic, saphenous, obturator, facial, supraorbital, lingual and lingual nerve [1]. Injury is usually caused when a nerve is subjected to stretch, ischemia or compression at the time of surgery. Stretch or prolonged compression my lead to end-neural edema, Schwann cell damage and/or demyelination, depending upon the duration [1]. In an awake patient poor positioning or compression leads to discomfort prompting to change in position to relieve the symptoms. In an anaesthetized patient this protective mechanism is abolished predisposing patient to nerve injury [1].

**Figure 3:**
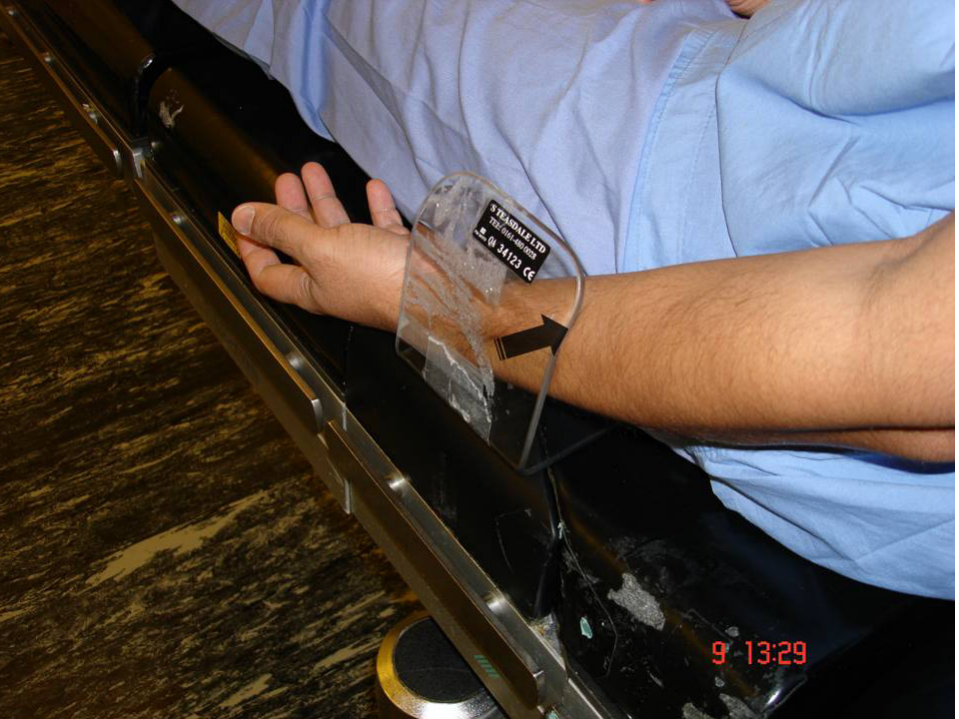
**Showing the possible point of compression of superficial radial nerve (SRN) in the arm-rest**. The arrow indicates to the interface between the skin and arm-rest, the point of maximum pressure.

Isolated injuries of SRN have been described due to direct injury during operation, venous cannulation, compression by bony lesion or soft tissue swelling [3-5]. Intraoperative positioning related injury of SRN has never been described in literature, and to our knowledge this is the first reported case of this type. In our case SRN was injured because of the prolonged compression of forearm against a poorly padded arm side support, as simulated in Figures 1, 2, 3.

Patient should be assessed preoperatively to search for predisposing factors such as diabetes, peripheral vascular disease, malnutrition, patient built etc. to minimize the risk of injury. Patient with any predisposing risk factors should be recognized and extra precaution should be taken [1]. Thankfully, most of the injuries recover completely over a period of time but is not true in all the cases. In our case patients symptoms markedly improved over period of time but she was left with hypoesthesia in dorsum of first web space.

## Conclusion

Positioning nerve injury is known to every surgeon and, despite the robust preventable measures in place, it still continues to occur from time to time. Detailed history taking and thorough preoperative assessment is vital in order identify the high-risk patient to place extra safety measures in the group. When it occurs is it imperative to identify it early, follow the patients up for recovery and refer them to appropriate specialist for necessary treatment.

## Abbreviations

SRN: Superficial Radial Nerve.

## Competing interests

The authors declare that they have no competing interests.

## Authors' contributions

VS Operating surgeon, collected clinical details including photographs, summarised the case history and prepared first draft. PS Conducted a literature search, design and formatting of final manuscript, preparation of final manuscript including grammar, punctuation and style. He also verified the authenticity of scientific content. AA Helped in conducting the literature search and extracting the papers from library and internet. He also contributed in preparation of electronic images and electronic formatting of manuscript.

## Consent

A fully informed written consent was obtained from the patient for the publication of this case report and accompanying images. A copy of the written consent is available for review by the Editor-in-Chief of this journal.
